# Genetic Analysis of Adaptive Traits in Spring Wheat in Northeast China

**DOI:** 10.3390/life14020168

**Published:** 2024-01-24

**Authors:** Hongji Zhang, Yuyao Li, Wenlin Liu, Yan Sun, Jingquan Tang, Jingyu Che, Shuping Yang, Xiangyu Wang, Rui Zhang

**Affiliations:** 1 Crop Resources Institute, Heilongjiang Academy of Agricultural Sciences, Harbin 150086, China; yzlwl12315@163.com (W.L.); sunyan720722@sohu.com (Y.S.); tangjingquan2020@163.com (J.T.); hljszwxh@163.com (S.Y.); 18845870508@163.com (X.W.); 2 Heilongjiang Academy of Agricultural Sciences, Harbin 150086, China; liyuyao0126@163.com; 3 Keshan Branch of Heilongjiang Academy of Agricultural Sciences, Qiqihar 161600, China; cryu1122@163.com; 4 Institute of Forage and Grassland Sciences, Heilongjiang Academy of Agricultural Sciences, Harbin 150086, China; zr0705@126.com

**Keywords:** adaptive traits, association analysis, candidate gene, marker-assisted selection, *Triticum aestivum* L.

## Abstract

The dissection of the genetic architecture and the detection of the loci for adaptive traits are important for marker-assisted selection (MAS) for breeding. A spring wheat diversity panel with 251 cultivars, mainly from China, was obtained to conduct a genome-wide association study (GWAS) to detect the new loci, including the heading date (HD), maturating date (MD), plant height (PH), and lodging resistance (LR). In total, 41 loci existing in all 21 chromosomes, except for 4A and 6B, were identified, and each explained 4.3–18.9% of the phenotypic variations existing in two or more environments. Of these, 13 loci are overlapped with the known genes or quantitative trait loci (QTLs), whereas the other 28 are likely to be novel. The 1A locus (296.9–297.7 Mb) is a multi-effect locus for LR and PH, whereas the locus on chromosome 6D (464.5–471.0 Mb) affects both the HD and MD. Furthermore, four candidate genes for adaptive traits were identified, involved in cell division, signal transduction, and plant development. Additionally, two competitive, allele-specific PCR (KASP) markers, *Kasp_2D_PH* for PH and *Kasp_6D_HD* for HD, were developed and validated in another 162 spring wheat accessions. Our study uncovered the genetic basis of adaptive traits and provided the associated SNPs and varieties with more favorable alleles for wheat MAS breeding.

## 1. Introduction

Common wheat is cultivated on about 210 million hectares worldwide and provides nearly 1/5 of the total energy input for the whole world’s population. Spring wheat accounts for approximately 14% of the total wheat planting area and 10% of the total crop yield in China, primarily located in the northwest and northeast regions. Major challenges faced in production include low yields per unit area and relatively poor adaptability, yield stability, and quality. Improving the varieties of spring wheat is a crucial approach to enhancing its competitiveness. Adaptive improvement is an important and challenging goal in wheat breeding [1]. The northeast region of China, like the Heilongjiang and Jilin provinces, is the most important spring wheat-producing area [2,3]. However, wheat production is facing the threat of a bottleneck for the adaptive potential of the introduced new germplasm, which is important for the improvement in genetic diversity [3,4]. Adaptive traits are complex and seriously influenced by environmental and genetic factors.

Adaptive traits include the heading date (HD), maturing date (MD), plant height (PH), and lodging resistance (LR) [5,6]. The PH is a critical agronomic trait of wheat, contributing significantly to plant architecture, and it is directly associated with resistance to lodging, yield, and harvest index, making it one of the key factors for high and stable wheat production [5]. In the 1960s, the utilization of dwarfing genes, such as *Rht1* and *Rht2*, substantially enhanced wheat lodging resistance and yield per unit area, triggering the Green Revolution in agricultural production. Subsequently, a series of dwarfing genes were identified and mapped from mutational and natural variant populations [5,6]. The HD is among the main agronomic traits of wheat, influencing yield, quality, ecological adaptability, and stress resistance. The HD is closely related to external factors, such as temperature and light, and it is often dramatically affected during climate changes and adverse weather conditions, impacting wheat production safety [7,8,9]. The timing of heading plays a crucial role in wheat breeding, selection, and cultivation across regions. Lodging is another heavy threat to wheat production, not only disrupting the canopy structure and impairing photosynthesis but also reducing the transport efficiency of water and photosynthetic products, which affects the grain-filling rate and duration. Currently, fostering LR is one of the essential breeding objectives in major wheat-growing regions globally [7,10]. Since the 1960s, the widespread use of dwarfing genes has substantially improved lodging resistance and boosted yields in wheat varieties worldwide. However, an excessively low PH can also reduce the above-ground biomass, impacting photosynthesis and subsequently lowering yields [2]. Therefore, it is crucial to maintain a suitable PH while enhancing LR further in wheat-breeding processes [1,2,3].

Marker-assisted selection (MAS) plays an important role in breaking through the bottleneck of wheat breeding and is an effective approach for improving the adaptive potential [3,5,7]. The number of available genes and correcting functional/closely related markers is important for the effectiveness and reliability of molecular breeding. However, to date, over 70 genes have been cloned, but only about 20 loci have been associated with adaptive traits in common wheat [8,9,10]. Although over 60 loci have been reported by GWAS or traditional linkage mapping [8,10], the genes or QTLs are still not enough to accelerate adaptive wheat breeding, particularly for spring wheat, due to the complexity of the genetic mechanism and the lack of available genes/loci. Compared with SSRs and InDels, single-nucleotide polymorphisms (SNPs) are richer and higher coverage. Now, genotyping for SNPs plays a vital role in the field of genes/loci identification using linkage mapping or association mapping [5,11]. In the last decade, the 55 K, 35 K, 90 K, and 660 K wheat SNP arrays have been developed and are gradually replacing SSRs and diversity arrays technology (DArT), and they are widely applied in the linkage or association mapping for grain yield, end-use quality, disease resistance, and abiotic stress tolerance [7,12]. Both bi-parental mapping and GWAS are two main ways to uncover the genetic basis of agronomical traits [11]. GWAS is based on linkage disequilibrium (LD) and offers an effective approach to genetic analysis [11]. Compared with traditional bi-parental linkage analysis, association mapping uses natural accessions and bypasses the time (5–8 years) and cost of developing bi-parental populations [7]. Furthermore, GWAS could be used for analysis using the same genotype data [11], whereas the traditional linkage analysis focused on specific traits. GWAS is widely used in genetic analysis to determine aspects such as grain yield, disease resistance, progressing quality, and abiotic stress [7,12].

The northeast spring wheat region is the main region of spring wheat production in China. The main challenges faced in spring wheat production include low yields per unit area, poor adaptability, and low yield and quality stability. Due to limited research on wheat adaptability traits, the currently discovered loci and genes are far from meeting the demands of molecular breeding for wheat. Exploring the adaptive loci or genes of spring wheat and improving its varieties are key approaches to enhancing its competitiveness. In the present study, a total of 251 accessions, mainly from the northeast of China (1930–2020), were selected for study to achieve the following purposes: (1) identify the loci and corresponding candidate genes of adaptive traits and (2) develop available competitive allele-specific PCR (KASP) markers for the improvement in wheat adaptive-related traits. To explore the genetic mechanisms underlying the adaptive traits of spring wheat, it is essential to identify usable genetic loci and develop applicable markers. Our research is of great significance in enhancing wheat adaptability through the utilization of molecular markers in assisted breeding.

## 2. Materials and Methods

### 2.1. Plant Materials and Field Trials

All the 251 wheat accessions, mainly from Heilongjiang and Jilin provinces of China (Table S1), were planted at Harbin and Keshan of Heilongjiang province during the 2018–2019 and 2019–2020 cropping seasons. In addition, another panel with 162 wheat cultivars primarily originating from the Northwest Spring Wheat Region also assessed the related adaptive traits to validate the effectiveness of the developed KASP markers. All 162 accessions were planted at Harbin and Keshan of Heilongjiang province during the 2020–2021 and 2021–2022 cropping seasons. A randomized complete block design with three replicates was employed in both locations. This featured four 2.0 m rows 20 cm apart and with 40 seeds in each row.

Harbin is characterized by a mid-temperate continental monsoon climate, with long winters and short summers. The annual average temperature of Harbin is 5.6 °C, with the highest monthly average temperature at 23.6 °C and the lowest monthly average temperature at −15.8 °C. The annual average precipitation is 423 mm, mainly concentrated between June and September, with a frost-free period of approximately 168 days. Keshan of Heilongjiang province experiences a cold temperate continental monsoon climate, with an annual average temperature of 2.4 °C and effective accumulated temperatures of 2400 °C. The annual precipitation is around 499 mm, with a frost-free period of approximately 122 days. Rainfall in Keshan is concentrated in June, July, and August, and the annual average precipitation is about 500 mm.

### 2.2. Phenotyping and Statistical Analysis

Four adaptive traits were evaluated, including HD, MD, PH, and LR. Of these, HD represented as days from sowing to heading and recorded the date of half-spike emergence (generally, in Harbin, the sowing dates are around 25–28 March, while in Keshan, the sowing dates are around 1–3 April). Ten plants in each plot were randomly selected at physiological maturity to measure PH. MD means the wheat plants turn yellow and the grains reach the end of wax ripening. The LR was recorded across the entire grain-filling stage. The degree of lodging in wheat plants is classified into four levels: (1) level 0: all plants in the field remain upright without any tilting. (2) Level 1: all plants display a tilting at an angle between 0 and 15 degrees. (3) Level 2: all plants exhibit tilting at an angle ranging from 15 to 45 degrees. (4) Level 3: all plants experience tilting at an angle greater than 45 degrees. BLUP was calculated using the MIXED procedure in SAS v9.3 (http://www.sas.com (accessed on 13 January 2024)) as follows: y (phenotype) = X_b_ (environment) + Zu (genotype) + e (residual effect).

### 2.3. Population Structure and Linkage Disequilibrium

The diverse panel was genotyped using the wheat 55 K SNP array (missing data > 20% and minor allele frequency < 0.05). The physical positions of SNPs were according to Chinese spring reference genome (IWGSC v1.1). To uncover the kinship and population structure, structure, principal component analysis (PCA) and neighbor jointing (NJ) were analyzed by Structure v2.3.4 and Tassel v5.0 [13,14] as reported by Li et al. [15]. The LD decay analysis was also reported by Li et al. [15]. The broad sense heritability (*H*_b_^2^) was conducted according to *H*_b_^2^ = σ_g_^2^/(σ_g_^2^ + σ_ge_^2^/r + σ_ε_^2^/re). Of these, σ_g_^2^, σ_ge_^2^, and σ_ε_^2^ represent the genotype, genotype × environment, and residual error, respectively; r and e indicate the number of replicates (environment) and environments, respectively.

### 2.4. Association Mapping and the Identification of Candidate Genes

Mixed linear model (MLM, PCA + K model) was used for association mapping in consideration of kinship matrix and population structure using Tassel V5.0. An adjusted −log_10_ (*p*-value) ≥ 3.0 was regarded as threshold for significant loci. Manhattan and Q-Q plots were drawn by the CMplot package (https://github.com/YinLiLin/CMplot (accessed on 13 January 2024)) based on R language (R 3.6.5). To identify candidate genes, the associate SNP flanking sequences were used in BLASTx against the NCBI and ENA databases. In addition, the Chinese spring (IWGSC v1.1, https://wheat.pw.usda.gov/GG3/ (accessed on 13 January 2024)) annotation was used as the reference.

### 2.5. Establishment and Verification of KASP Markers

Six SNPs with consistent and stable effects were subsequently converted into KASPs [5] and designed by the PolyMarker (http://www.polymarker.info/ (accessed on 13 January 2024)). We utilized the PHERA starplus SNP (BMG Labtech GmbH, Ortenberg, Germany) to interpret the 384-well plates and identify the genotype analysis using KlusterCaller software (LGC, Hoddesdon, UK). Each KASP was critically validated by 162 cultivars, mainly from the Northwest and Northeast spring wheat production regions of China.

### 2.6. Identification of the Candidate Genes of Adaptive Related Traits

To identify the candidate genes that might be functioning within the QTL for adaptive traits, high-confidence genes located within the LD block encompassing the peak SNP (approximated to ±3.0 Mb) of each locus were detected based on the IWGSC v1.1 annotation. Among those, we ruled out hypothetical transposon and retrotransposon proteins and considered only genes bearing SNPs within their coding region as confidence candidate genes for subsequent investigations.

## 3. Results

### 3.1. Phenotypic Evaluation

Significant and continuous variations of HD, MD, PH, and LR were exhibited in the diverse panel (Table S1; Figure S1). The mean values of HD, MD, PH, and LR were 83.1 d (72.4–88.5 d), 108.7 d (105.2–112.8 d), 108.1 cm (76.5–134.8 cm), and 0.23 (0.02–3.0). LR showed significant (*p* < 0.01) and positive correlations with PH (r = 0.65). MD showed significant (*p* < 0.01) and positive correlations with HD (r = 0.36). Highly significant effects (*p* < 0.01) of genotypes, environments, and genotype × environment were shown by ANOVA on all adaptive traits (Table 1). The broad sense heritability (*H*_b_^2^) estimated for HD, MD, PH, and LR were 0.54, 0.50, 0.65, and 0.47, respectively, indicating that all four adaptive traits were mainly determined by genetics.

### 3.2. Population Structure and LD Decay Analysis

A total of 52,503 polymorphic SNPs were used for association mapping (0.273 Mb per marker) [15] (Figure S2). All the 251 accessions mainly belonged to three subgroups, and each contained 126 (Heilongjiang ranged from the 1950s to 1980s, mainly including Keshan, Longchun, Longfumai, Hechun, and Longmai series), 75 (Heilongjiang ranged from the 1990s to 2010s, mainly including Kechun, Beimai, and Longfu series), and 50 varieties (from Jilin and foreign counties, including Poland, Canada, Australia, Japan, and Mexico) (Table S1, Figure S3). In addition, LD decay analysis indicated that the average LD decay distance for the whole genome was about 8 Mb [15] (Figures S3 and S4).

### 3.3. Genome-Wide Association Studies

**Figure 1:**
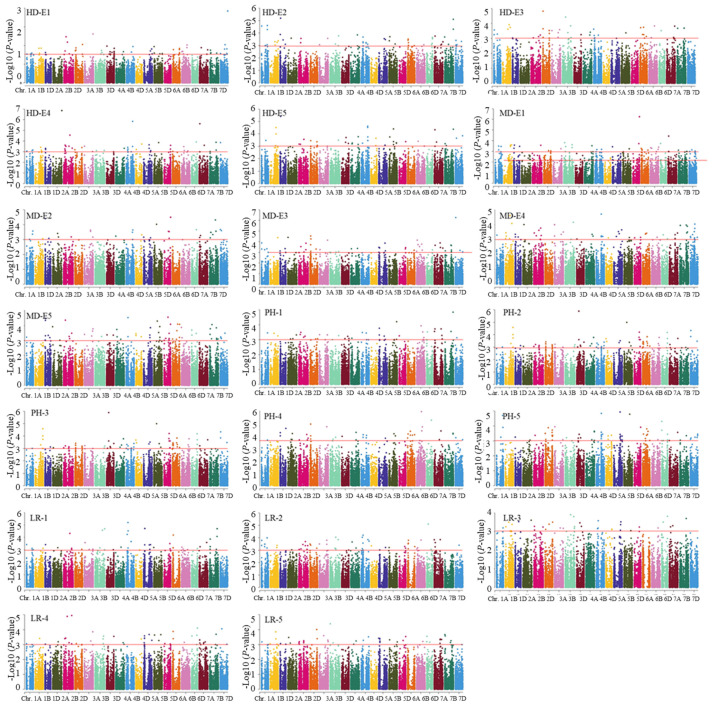
Manhattan plot of adaptive related traits in 251 wheat accessions analyzed by the mixed linear model (MLM) in Tassel v5.0. HD: heading date; LR: lodging resistance; MD: maturing date; PH: plant height. The numbers 1, 2, 3, 4, and 5 indicate Harbin 2018, Harbin 2019, Keshan 2018, Keshan 2019, and the best linear unbiased prediction (BLUP).

In this study, an MLM model featuring a combination of PCA and a kinship matrix was implemented to conduct the association analysis. In total, 41 loci associated with wheat adaptive traits were observed across two or more environments (Table 2 and Table S2, Figure 1 and Figure S5). Among these, it was found that 10 loci associated with LR were distributed on 1A, 1D, 3A, 3D, 5A, 5B, 5D, 6A, and 6D, and each explained 4.3–11.2% of the phenotypic variations. In addition, eight loci on chromosomes 1D, 2A, 2D, 4D, 5B, 6A, 6D, and 7D were affiliated with the MD, and each explained 4.4% to 10.8% of the total phenotypic variations. Furthermore, 15 loci linked to PH were detected across 1A, 1D, 2B, 2D (2), 3A, 3B, 4D, 5B, 5D, 6A, 7A, 7B, and 7D (2) chromosomes, and each of these contributed 5.6–10.7% of the phenotypic variances. Lastly, 10 loci associated with HD were found to be distributed on chromosomes 1A, 1B, 2A, 2D, 3A, 4A, 5A, 5B, 6A, and 6D. These loci demonstrated explanations of 5.7–18.9% of the phenotypic variances. Among these, two 1A (296.9–297.7 Mb) showed pleiotropic effects for LR and PH, while another locus on chromosome 6D (464.5–471.0 Mb) concurrently controlled both HD and MD.

### 3.4. Candidate Genes

Seven candidate genes related to adaptive traits were identified (Table 3). Of these, E3 ubiquitin-protein ligase-like protein *TraesCS2A01G248400* and *TraesCS1A01G164400* were located in the loci of 1A (297.7 Mb) and 2A (379.9–410.9 Mb). Additionally, *TraesCS2D01G591000,* encoding serine/threonine-protein kinases, was identified at the locus of 2D (643.7–650.1 Mb). Simultaneously, *TraesCS1A01G343000* and *TraesCS6A01G356200* emerged as candidate genes that encoded the ATP-binding cassette (ABC) transporters, specifically corresponding to 1A (513.0 Mb) and 6A (564.4–573.5 Mb). In addition, two other candidate genes associated with distinctive protein families were identified. The calcium-dependent lipid-binding (CaLB domain) family gene *TraesCS7A01G520000* was linked to the 7A (720.0–722.3 Mb), while the B3 transcription factor family protein *TraesCS1B01G392000* was identified for the 1B locus (676.2 Mb).

### 3.5. KASP Validation

To provide available KASP markers for wheat breeding, six SNPs manifesting stable or consistent effects were selected for conversion to KASP markers. Regrettably, during the conversion process, four SNPs, including *AX-111096297* (2D 33.0 Mb) for PH, *AX-111614560* (5A 553.4 Mb) for HD, *AX-109360059* (1A 277.1 Mb) for LR, and *AX-110672099* (6A 594.8 for MD), could not be transformed into KASP markers. Furthermore, the genotyping of these SNPs was not accomplished successfully. *AX-109836946* (2D 33.0 Mb) for PH and *AX-110918412* (6D 464.9 Mb) for HD were successfully converted into KASP markers (Table S3) and then tested on a set of 162 diverse cultivars. For *Kasp_2D_PH*, the favorable allele (GG) accounted for 70.5% and had a mean PH of 74.0 cm, significantly lower than the unfavorable allele (CC), which accounted for 22.1% with a mean PH of 78.5 cm (*p* = 0.05) (Table S4). Similarly, for *Kasp_6D_HD*, the favorable allele (CC), making up 27.6% with a mean HD of 69.9 d, significantly outperformed the unfavorable allele (AA) that accounted for 64.4% and had a mean HD of 72.0 d (*p* = 0.05) (Table 4 and Table S4). Therefore, these markers provide credible insights into the genetic basis defining important traits of wheat, opening possibilities for further research and application in crop improvement programs.

## 4. Discussion

PH is a critical component of plant architecture, directly related to the LR, yield, harvest index, and other important traits. Appropriate PH is an important consideration in the introduction of new wheat varieties. LR, a crucial element of wheat adaptive traits, is a significant threat to wheat production. PH directly impacts the LR of the plant. However, a lower PH can also decrease the yield in wheat production. Therefore, it is more important to improve the LR on the basis of a certain PH. The heading and ripening stages are closely related to environmental factors, such as temperature and light, yield, quality, ecological adaptability, and resistance to adverse conditions of wheat. These factors hold significant importance for regional introduction and selection. Adaptive traits are an important reference for the introduction and selection of wheat and the foundation of high yield and stable production. Therefore, identifying the loci of adaptive traits and developing available molecular markers have significant implications for high-yielding and stable wheat production.

### 4.1. Twenty-Eight Novel Loci for Adiptive Traits in Common Wheat Were Identfied

SNP arrays were developed based on next-generation sequencing technology, making SNP markers in large quantities and with high throughput [16]. In the present study, 52,503 SNP markers were used in constructing a high-density physical map, with an average density of 0.273 Mb per marker. However, the average LD for the whole genome is 8 Mb. The high-density of SNP markers used in the present study ensured numerous markers in each LD and revealed high efficiency in significant loci identification [15]. The 251 accessions belonged to three subgroups, which were largely consistent with geographic origins. Previous studies have reported some loci for adaptive traits in common wheat. In this study, association mapping of adaptive traits was conducted. In total, 10, 8, 15, and 10 loci were detected for LR, MD, PH, and HD, respectively, and each explained 5.6–17.8% of the phenotypic variances.

### 4.2. Heading Date

The HD is an important adaptive trait for common wheat. Li et al. [3] have reported eight loci for HD on chromosomes 2A (3), 2B, 5B, 7A (2), and 7B and accounted for 6.6–13.1% of the PVE, respectively. Of these, two loci on chromosome 2A and 5B coincided with the loci identified in our study (2A: 209.2 Mb, and 5B: 521.0–521.7 Mb). Moreover, Le Gouis et al. [17] have reported two DArT markers, *wPt-1499* and *wPt-1409,* to be significantly associated with HD on chromosomes 2A and 5A, respectively. These two markers are close to the HD loci of (2A: 209.2 Mb and 5A: 553.0–553.4 Mb), respectively, based on the consensus linkage map. On chromosome 1B, we detected a locus at 676.2–676.7 Mb, which overlapped with the LD decay distance of a locus for flowering time identified by Luján et al. [18]. On the other hand, Luján et al. [18] showed a locus associated with extra-early flowering time and close to the loci in this study (596.2–601.5 Mb). As the majority of varieties are from the northeast of China with similar vernalization and photoperiod characteristics, no significant associations were detected for HD with the genes *Vrn-A1* (5A), *Vrn-B1*, *Vrn-D1* (5D) [19], and *Ppd-B1* (2B) [20], indicating that this gene did not appear to affect the heading time in this panel. Also, it is possible that the potentially high epistatic interactions among vernalization genes caused difficulties in the identification of minor effects genes.

### 4.3. Maturing Date

MD is a complex trait and difficult to evaluate. Until now, only a few reports have focused on the genetic basis of MD. Xiang et al. [21], Semagn et al. [22], and Zhang et al. [23] have reported a series of loci for wheat HD and MD. However, no overlapping has been identified by Maccaferri et al. [24] and IWGSC v1.1. Thus, we infer all the seven loci for MD were novel.

### 4.4. Plant Height

Plant height is an important factor for lodging resistance. We identified 15 loci for PH in this study and explained 7.1–10.7% of the phenotypic variances, respectively. Li et al. [3] identified 14 loci for PH on chromosomes 1A, 1B (2), 2A (2), 3A, 3B, 4D, 5A (2), 5B, 6B (2), and 7A, and each explained 6.7–30.8% of the phenotypic variances, respectively. Of these, the loci on chromosome 1A, 3B and 5B were overlapped with the region 1A (513.0 Mb), 3A (69.3–70.7 Mb), and 5B (571.5–593.5 Mb) identified in our study. *Rht-D1b* is widely spread in wheat [25,26]. The PH locus (26.0–28.8 Mb on chromosome 4D) is at the same position as *Rht-D1*, indicating the effect on PH is from *Rht-D1b*, and it is the same as the QTL or loci reported by Gao et al. [25], Sun et al. [27], and Li et al. [6]. Cui et al. [9] identified that a QTL for PH on chromosome 3A is close to the loci (69.3–70.7 Mb) identified in our study. Some known PH genes, such as *Rht25* (6A: 144.0–148.3 Mb) and *Rht18/Rht14/Rht24* (6A: 413.73 Mb), have been identified on chromosome 6A, according to Luján et al. [18]. However, none of these overlapped with the loci identified in our study. Furthermore, in the case of PH, we did not find significant associations between the PH and the “green revolution” dwarfism genes *Rht-B1* and *Rht-D1* [28]. Although the panel presented a wide variation in PH, the collection is mainly composed of semi-dwarf elite germplasm, and the *Rht-B1* and *Rht-D1* genes are balanced.

### 4.5. Lodging Resistance

A series of studies have reported the loci for LR, which are related to the plant height, stem diameter, and lignin content. Piñera-Chavez et al. [29] identified 18 QTL for LR on chromosomes 1D, 2B, 2D, 3A, 3B, 4A, 4D, 5B, and 6B. Of these, the loci on chromosome 1D overlapped with the loci 1D (416.6–421.9 Mb) identified in this study. Moreover, Li et al. [3] also identified 14 loci for PH on chromosomes 1A, 1B (2), 2A (2), 3A, 3B, 4D, 5A (2), 5B, 6B (2), and 7A. Of these, the loci on chromosome 3A and 5B overlapped with the loci in this study (3A: 619.5–6277 Mb; 5B: 420.1–424.2 Mb). The loci (3A: 619.5–6277 Mb) also coincided with the loci reported by Cui et al. [30]. Furthermore, Dreccer et al. [31], Song et al. [32], and Blackburn et al. [33] identified a series of loci for LR in common wheat. However, no overlapping was identified with this study, according to Maccaferri et al. [24] and the Chinese spring reference genome. Thus, the seven other loci may be novel.

In conclusion, the loci for LR on chromosome 1D (416.6–421.9 Mb), 3A (619.5–627.7 Mb), and 5B (420.1–424.2 Mb); the loci for PH on chromosome 1A (513.0–513.0 Mb), 3A (69.3–70.7 Mb), 4D (26.0–28.8 Mb), 5B (571.5–593.5), and 6A (564.4–573.5); and the loci for HD on chromosome 1B (676.2–676.7 Mb), 2A (209.2–415.3 Mb), 3A (596.2–601.5 Mb), 5A (553.0–553.4 Mb), and 5B (21.0–521.7 Mb) overlapped with previous studies. The other loci were considered as new loci. The stable loci validated by both GWAS and QTL mapping between our studies and previous studies indicated that they are widespread in diverse varieties.

### 4.6. Candidate Gene Analysis

In total, seven candidate genes were identified (Table 3). For the loci on chromosome 1A (297.7 Mb) and 2A (379.9–410.9 Mb), two candidate genes were identified that encoded the E3 ubiquitin-protein ligase-like protein, which plays an important role in the plant growth and development [34]. For 1A (513.0 Mb) and 6A (564.4–573.5 Mb), *TraesCS1A01G343000* and *TraesCS6A01G356200* were identified, which both encoded the ABC transporter. The ABC transporter has been reported to be involved in synchronizing plant growth. *TraesCS2D01G591000* encoded serine/threonine-protein kinases that were detected in the LD decay of the loci on chromosome 2D (643.7–650.1 Mb), which plays crucial roles in cell-cycle progression, flower formation, and signal transduction, according to Sánchez-Martín et al. [35]. The CaLB family (*TraesCS7A01G520000*) was identified on the loci 7A (720.0–722.3 Mb). The CaLB domain in plants is involved in various signaling pathways and plays a critical role in root growth and development [36]. The B3 transcription factor family protein (*TraesCS1B01G392000*) was identified as the candidate gene for the loci on chromosome 1B (676.2 Mb). The B3 transcription factor family is involved in plant growth and development, flowering, and vernalization responses in crop plants [37].

### 4.7. Potential Implications on Wheat Breeding

Previous studies indicated that significant additive effects were reported between adaptive traits and the number of favorable alleles, and pyramiding favorable alleles will improve adaptive traits effectively [6]. The loci with consistent and pleiotropic effects should be efficient for MAS breeding. Those loci validated by previous studies indicated the widespread varieties and could be applied in further study. KASP offers a cost-effective and flexible way to achieve MAS. In this study, *Kasp_2D_PH* and *Kasp_6D_HD* were successfully developed based on tightly linked SNPs and proved to be effective and valuable tools for MAS in wheat breeding with 162 cultivars. Additionally, accessions with better adaptive traits suitable for the local growing conditions, production requirements, and appropriate agronomic traits (such as Jichun 132, Jichun 201, Jichun 147, Jichun 157, and Jichun 158 with higher LR; Kenda 1, Xinshuguang 5, Hemai 3, Liaochun 2, and Shen 68–71 with early maturation; KeFeng 4, Kenda 1, Xinshuguang 5, Nonglin 45, and Jianada 13 with higher PH; and Liaochun 10, Mexico-1, Shen 68–71, Kenda 1, and Liaochun 2 with early HD) could be recommended as parental lines for wheat breeding.

## 5. Conclusions

In this study, association mapping for HD, MD, PH, and LR was conducted on 251 spring wheat accessions, and 28 new loci were identified. Four candidate genes involved in cell division, signal transduction, and plant development were detected. In addition, *Kasp_2D_PH* and *Kasp_6D_HD* for improved plant height and heading date were developed and can be utilized in wheat-breeding programs. Our study uncovers the genetic architecture of adaptive traits and provides available KASP markers and germplasm for wheat MAS breeding.

## Figures and Tables

**Table 1:** ANOVA analysis of the adaptive traits in 251 spring wheat accessions.

Source of Variation	df	*F*-Value			
		HD	MD	PH	LR
Genotypes	250	158.9 **	189.6 **	136.9 **	19.8 **
Environments	3	426.6 **	362.6 **	523.6 **	78.6 **
Replicates (nested in environments)	2	15.2 **	12.6 **	25.3 **	4.3 **
Genotypes × Environments	749	6.3 **	8.9 **	6.9 **	2.1 **
Error	1425				

HD: heading date; MD: maturing date; PH: plant height; LR: lodging resistance. ** indicates significance at 0.01 levels.

**Table 2:** Loci of adaptive traits in 251 spring wheat accessions by association analysis.

Trait	Chromosome	Representive SNP	Start (Mb)	End (Mb)	R^2^		*p*-Value		Environment	Favorable Allele
					Minimum	Maximum	Minimum	Maximum		
LR	1A	*AX-111014967*	125.2	134.0	4.5	4.8	1.9 × 10^−5^	3.9 × 10^−5^	E1, E2, BLUP	A
LR	1A	*AX-110910455*	275.1	282.2	6.8	7.2	8.3 × 10^−7^	5.8 × 10^−6^	E1, E2, BLUP	C
LR	1D	*AX-111722231*	416.6	421.9	6.5	7.1	4.0 × 10^−7^	1.6 × 10^−5^	E1, E2, BLUP	C
LR	3A	*AX-109947801*	619.5	627.7	4.5	4.6	2.9 × 10^−5^	5.4 × 10^−5^	E1, E2, BLUP	C
LR	3D	*AX-95160344*	512.3	517.8	5.2	5.3	3.4 × 10^−6^	5.6 × 10^−6^	E1, E2, BLUP	G
LR	5A	*AX-109934392*	535.5	540.0	4.3	4.6	4.5 × 10^−5^	3.9 × 10^−5^	E1, E2, E4, BLUP	G
LR	5B	*AX-109731422*	420.1	424.2	6.0	6.3	3.0 × 10^−6^	8.8 × 10^−6^	E1, E2, E4, BLUP	G
LR	5D	*AX-109351618*	9.5	11.9	4.9	11.2	5.1 × 10^−9^	1.9 × 10^−5^	E1, E2, E4, BLUP	G
LR	6A	*AX-111533379*	681.1	684.5	4.4	4.9	6.1 × 10^−5^	7.3 × 10^−5^	E1, E2, E4, BLUP	G
LR	6D	*AX-109643661*	7.4	10.7	4.4	4.8	4.6 × 10^−5^	5.1 × 10^−5^	E1, E2, E4, BLUP	G
MD	1D	*AX-110001111*	16.1	19.3	5.7%	7.1%	1.7 × 10^−4^	8.3 × 10^−4^	E1, E2, E4, BLUP	G
MD	2A	*AX-110505632*	379.9	410.9	5.6%	7.8%	9.3 × 10^−5^	9.7 × 10^−4^	E1, E3	G
MD	2D	*AX-108981520*	643.7	650.1	5.4%	10.8%	6.7 × 10^−6^	9.8 × 10^−4^	E2, BLUP	A
MD	4D	*AX-111354892*	14.7	15.4	4.4%	7.1%	2.0 × 10^−5^	7.1 × 10^−4^	E1, E3, E2, E4, BLUP	C
MD	5B	*AX-108905558*	532.8	533.0	5.8%	7.6%	6.9 × 10^−5^	8.8 × 10^−4^	E1, E3, E2, BLUP	C
MD	6A	*AX-110672099*	594.8	599.8	6.2%	10.0%	4.2 × 10^−6^	5.1 × 10^−4^	E1, E3, E2, E4, BLUP	C
MD	6D	*AX-95088462*	470.3	471.0	6.2%	8.5%	3.0 × 10^−5^	4.2 × 10^−4^	E1, E3, E2, E4, BLUP	G
MD	7D	*AX-110495278*	104.6	104.9	5.9%	7.8%	3.0 × 10^−4^	9.0 × 10^−4^	E1	G
PH	1A	*AX-111018634*	513.0	513.0	5.8%	8.9%	2.3 × 10^−5^	8.3 × 10^−4^	E1, E3, E2, E4, BLUP	G
PH	1D	*AX-110483115*	48.1	58.1	5.6%	9.8%	8.6 × 10^−6^	9.6 × 10^−4^	E2, E4, BLUP	G
PH	2B	*AX-110481288*	439.2	439.5	6.1%	8.8%	2.4 × 10^−5^	5.5 × 10^−4^	E1, E3, E1, E4, BLUP	C
PH	2D	*AX-111031463*	19.2	22.3	5.8%	7.3%	1.4 × 10^−4^	7.8 × 10^−4^	E1, E3, E2, E4, BLUP	C
PH	2D	*AX-111096297*	33.0	35.7	5.9%	10.7%	5.9 × 10^−6^	6.7 × 10^−4^	E1, E3, E1, E2, E4, BLUP	C
PH	3A	*AX-111570251*	69.3	70.7	6.0%	8.3%	4.8 × 10^−5^	6.1 × 10^−4^	E1, E3, E4, BLUP	C
PH	3B	*AX-109500452*	506.9	506.9	5.7%	8.5%	4.9 × 10^−5^	9.8 × 10^−4^	E1, E3, E1, E2, E4, BLUP	A
PH	4D	*AX-109908071*	26.0	28.8	6.0%	6.8%	3.4 × 10^−4^	6.8 × 10^−4^	E1, E3, E1, E2, E4, BLUP	A
PH	5B	*AX-110384536*	571.5	593.5	6.1%	8.0%	5.7 × 10^−5^	5.7 × 10^−4^	E1, E3, E1, E2, E4, BLUP	T
PH	5D	*AX-110036274*	562.0	562.7	5.8%	7.4%	1.2 × 10^−4^	7.5 × 10^−4^	E1, E3, E1, E2, E4, BLUP	G
PH	6A	*AX-89610547*	564.4	573.5	5.7%	7.5%	1.2 × 10^−4^	9.5 × 10^−4^	E1, E3, E2, BLUP	G
PH	7A	*AX-109036056*	720.0	722.3	5.8%	7.1%	2.2 × 10^−4^	8.9 × 10^−4^	E1, E3, E1, BLUP	G
PH	7B	*AX-94434165*	0.1	0.1	5.8%	7.4%	1.3 × 10^−4^	7.5 × 10^−4^	E1, E3, E1, E4, BLUP	G
PH	7D	*AX-110052150*	71.6	76.9	5.7%	9.6%	9.6 × 10^−6^	9.3 × 10^−4^	E1, E3, E2, E4, BLUP	G
PH	7D	*AX-94842881*	376.1	379.8	6.0%	7.1%	1.8 × 10^−4^	7.1 × 10^−4^	E1, E3, E1, E2, E4, BLUP	G
HD	1A	*AX-111187227*	297.7	297.7	6.0%	7.6%	1.1 × 10^−4^	6.6 × 10^−4^	E1, E3, E2, BLUP	G
HD	1B	*AX-111469159*	676.2	676.7	5.8%	8.8%	2.6 × 10^−5^	9.4 × 10^−4^	E1, E3, E2, BLUP	C
HD	2A	*AX-111155549*	209.2	415.3	6.1%	6.6%	6.7 × 10^−4^	6.9 × 10^−4^	E1, E3, E2, BLUP	C
HD	2D	*AX-110203406*	15.7	56.6	6.0%	18.9%	6.3 × 10^−10^	6.7 × 10^−4^	E1, E3, E1, E2, E4, BLUP	C
HD	3A	*AX-111055674*	596.2	601.5	5.7%	7.7%	1.4 × 10^−4^	9.6 × 10^−4^	E1, E3, E1, E2, E4, BLUP	C
HD	4A	*AX-108880805*	659.2	660.1	5.7%	7.8%	1.5 × 10^−4^	9.4 × 10^−4^	E1, E2, BLUP	C
HD	5A	*AX-108774016*	553.0	553.4	5.8%	9.5%	1.0 × 10^−5^	8.2 × 10^−4^	E1, E2, E4, BLUP	G
HD	5B	*AX-111138644*	521.0	521.7	5.7%	6.8%	3.1 × 10^−4^	9.4 × 10^−4^	E1, E2, E4, BLUP	G
HD	6A	*AX-95073334*	425.8	431.2	6.0%	9.4%	1.1 × 10^−5^	7.3 × 10^−4^	E2, E4, BLUP	G
HD	6D	*AX-110918412*	464.9	465.3	5.7%	10.7%	4.4 × 10^−6^	9.0 × 10^−4^	E1, E3, E2, BLUP	C

HD: heading date; MD: maturing date; PH: plant height; LR: lodging resistance. E1, E2, E3, E4, and E5 indicate Haerbin 2018, Haerbin 2019, Keshan 2018, Keshan 2019, and the best linear unbiased prediction (BLUP).

**Table 3:** The details of the candidate genes of adaptive traits.

Candidate Gene	Chromosome	Start (bp)	End (bp)	Annotation
*TraesCS1A01G164400*	1A	296212311	296215426	E3 ubiquitin-protein ligase
*TraesCS1A01G343000*	1A	531717134	531722196	ATP-binding cassette transporter
*TraesCS1B01G392000*	1B	624970835	624972070	B3 transcription factor family
*TraesCS2A01G248400*	2A	371044868	371046934	E3 ubiquitin-protein ligase family
*TraesCS2D01G591000*	2D	646593035	646594771	Serine/threonine-protein kinase
*TraesCS6A01G356200*	6A	587551821	587553888	ATP-binding cassette transporter
*TraesCS7A01G520000*	7A	704387759	704388700	Calcium-dependent lipid-binding family

**Table 4:** Effects of *Kasp_4A_RL* and *Kasp_5D_RT* on RSA-related traits in the natural population.

Marker	SNP	Genotype	Number	Phenotype	*p*-Value
*Kasp_2D_PH*	*AX-111096297*	CC	36	78.5 cm (PH)	0.049 *
		GG	115	74.0 cm (PH)	
*Kasp_6D_HD*	*AX-110918412*	AA	105	72.0 d (HD)	0.023 *
		CC	45	69.9 d (HD)	

* Significant at *p* < 0.05.

## Data Availability

All datasets generated for this study are included in the article/supplementary material; further inquiries can be directed to the first author.

## Supplementary Materials

The following supporting information can be downloaded at: https://www.mdpi.com/article/10.3390/life14020168/s1, Figure S1: The histogram of the adaptive related traits in 251 spring wheat accessions. HD: heading date; MD: maturing date; PH: plant height; LR: lodging resistance. E1, E2, E3, E4, and E5 indicate Harbin 2018, Harbin 2019, Keshan 2018, Keshan 2019, and the best linear unbiased prediction (BLUP). Figure S2: Marker density on each chromosome of the GWAS panel genotyped using the wheat 55 K SNP array [15]. Different colors represent the corresponding number of SNPs within a 1 Mb distance on each of the 21 chromosomes (based on IWGSC 2.1); Figure S3. Population analysis of the 251 spring wheat accessions [15], (a) Delta K for structure analysis; (b) Population structure analysis; (c) Neighbor-joining (NJ) tree; (d) Principal components analysis (PCA) plots. Figure S4: The LD decay of the whole genome of the 251 accessions [15]. Figure S5: Q-Q plot of adaptive traits in 251 wheat accessions analyzed by the mixed linear model (MLM) in Tassel v5.0. HD: heading date; MD: maturing date; PH: plant height; LR: lodging resistance. E1, E2, E3, E4, and E5 indicate Harbin 2018, Harbin 2019, Keshan 2018, Keshan 2019, and the best linear unbiased prediction (BLUP). Table S1: The details of the adaptive traits in 251 spring wheat accessions. HD: heading date; LR: lodging resistance; MD: maturing date; PH: plant height. The numbers 1, 2, 3, 4, and 5 indicate Harbin 2018, Harbin 2019, Keshan 2018, Keshan 2019, and the best linear unbiased prediction (BLUP). Table S2: The marker trait associations (MTAs) of related adaptive traits identified in this study. Table S3: The primers of Kasp_2D_PH and Kasp_6D_HD. Table S4: The genotype and the details for Kasp_2D_PH and Kasp_6D_HD in 162 wheat accessions.

## References

[1] Hyles J., Bloomfield M.T., Hunt J.R., Trethowan R.M., Trevaskis B. (2020). Phenology and related traits for wheat adaptation. Heredity.

[2] Li H., Zhou Y., Xin W., Wei Y., Zhang J., Guo L. (2019). Wheat breeding in northern China: Achievements and technical advances. Crop J..

[3] Li F., Wen W., Liu J., Zhang Y., Cao S., He Z., Rasheed A., Jin H., Zhang C., Yan J. (2020). Genetic architecture of grain yield in bread wheat based on genome-wide association studies. BMC Plant Biol..

[4] Wang Y., Hou J., Liu H., Li T., Wang K., Hao C., Liu H., Zhang X. (2019). TaBT1, affecting starch synthesis and thousand kernel weight, underwent strong selection during wheat improvement. J. Exp. Bot..

[5] Rasheed A., Wen W., Gao F., Zhai S., Jin H., Liu J., Guo Q., Zhang Y., Dreisigacker S., Xia X. (2016). Development and validation of KASP assays for genes underpinning key economic traits in bread wheat. Theor. Appl. Genet..

[6] Li F., Wen W., He Z., Liu J., Jin H., Cao S., Geng H., Yan J., Zhang P., Wan Y. (2018). Genome-wide linkage mapping of yield related traits in three Chinese bread wheat populations using high-density SNP markers. Theor. Appl. Genet..

[7] Liu J., He Z., Wu L., Bai B., Wen W., Xie C., Xia X. (2016). Genome-wide linkage mapping of QTL for black point reaction in bread wheat (*Triticum aestivum* L.). Theor. Appl. Genet..

[8] Chen Z., Cheng X., Chai L., Wang Z., Du D., Wang Z., Bian R., Zhao A., Xin M., Guo W. (2020). Pleiotropic QTL influencing spikelet number and heading date in common wheat (*Triticum aestivum* L.). Theor. Appl. Genet..

[9] Cui F., Li J., Ding A., Zhao C., Wang L., Wang X., Li S., Bao Y., Li X., Feng D. (2011). Conditional QTL mapping for plant height with respect to the length of the spike and internode in two mapping populations of wheat. Theor. Appl. Genet..

[10] Zhang L., Zhang H., Qiao L., Miao L., Yan D., Liu P., Zhao G., Jia J., Gao L. (2021). Wheat MADS-box gene TaSEP3-D1 negatively regulates heading date. Crop J..

[11] Zhu C., Gore M., Buckler E.S., Yu J. (2008). Status and prospects of association mapping in plants. Plant Genome.

[12] Quan X., Dong L.J., Zhang N., Xie C., Li H., Xia X., He W., Qin Y. (2021). Genome-wide association study uncover the genetic architecture of salt tolerance-related traits in common wheat (*Triticum aestivum* L.). Front. Genet..

[13] Pritchard J.K., Stephens M., Rosenberg N.A., Donnelly P. (2000). Association mapping in structured populations. Am. J. Hum. Genet..

[14] Breseghello F., Sorrells M.E. (2006). Association mapping of kernel size and milling quality in wheat (*Triticum aestivum* L.) cultivars. Genetics.

[15] Li Y., Tang J., Liu W., Yan W., Sun Y., Che J., Tian C., Zhang H., Yu L. (2021). The genetic architecture of grain yield in spring wheat based on genome-wide association study. Front. Genet..

[16] Wang S.C., Wong D., Forrest K., Allen A., Chao S., Huang B.E., Maccaferri M., Salvi S., Milner S.G., Cattivelli L. (2014). Characterization of polyploid wheat genomic diversity using a high-density 90000 single nucleotide polymorphism array. Plant Biotechnol. J..

[17] Le Gouis J., Bordes J., Ravel C., Heumez E., Faure S., Praud S., Galic N., Remoué C., Balfourier F., Allard V. (2012). Genome-wide association analysis to identify chromosomal regions determining components of earliness in wheat. Theor. Appl. Genet..

[18] Luján Basile S.M., Ramírez I.A., Crescente J.M., Conde M.B., Demichelis M., Abbate P., Rogers W.J., Pontaroli A.C., Helguera M., Vanzetti L.S. (2019). Haplotype block analysis of an Argentinean hexaploid wheat collection and GWAS for yield components and adaptation. BMC Plant Biol..

[19] Fu D., Szűcs P., Yan L., Helguera M., Skinner J.S., Von-Zitzewitz J. (2005). Large deletions within the first intron in Vrn-1 are associated with spring growth habit in barley and wheat. Mol. Genet. Genom..

[20] Díaz A., Zikhali M., Turner A.S., Isaac P., Laurie D.A. (2012). Copy number variation affecting the Photoperiod-B1 and Vernalization-A1 genes is associated with altered flowering time in wheat (*Triticum aestivum*). PLoS ONE.

[21] Xiang R., Semagn K., Iqbal M., Chen H., Yang R.C., Spaner D. (2021). Phenotypic performance and associated QTL of ‘Peace’×‘CDC Stanley’mapping population under conventional and organic management systems. Crop Sci..

[22] Semagn K., Iqbal M., Chen H., Perez-Lara E., Bemister D.H., Xiang R., Zou J., Asif M., Kamran A., N’Diaye A. (2021). Physical mapping of QTL associated with agronomic and end-use quality traits in spring wheat under conventional and organic management systems. Theor. Appl. Genet..

[23] Zhang J., Islam M.S., Zhao Y., Anwar M., Alhabbar Z., She M., Yang R., Juhasz A., Tang G., Chen J. (2022). Non-escaping frost tolerant QTL linked genetic loci at reproductive stage in six wheat DH populations. Crop J..

[24] Maccaferri M., Ricci A., Salvi S., Milner S.G., Noli E., Martelli P.L., Casadio R., Akhunov E., Scalabrin S., Vendramin V. (2015). A high- density, SNP-based consensus map of tetraploid wheat as a bridge to integrate durum and bread wheat genomics and breeding. Plant Biotechnol. J..

[25] Gao F., Ma D., Yin G., Rasheed A., Dong Y., Xiao Y., Xia X., Wu X., He Z. (2017). Genetic progress in grain yield and physiological traits in Chinese wheat cultivars of Southern Yellow and Huai Valley since 1950. Crop Sci..

[26] Gao F., Wen W., Liu J., Rasheed A., Yin G., Xia X., Wu X., He Z. (2015). Genome-wide linkage mapping of QTL for yield components, plant height and yield-related physiological traits in the Chinese wheat cross Zhou 8425B/Chinese Spring. Front. Plant Sci..

[27] Sun C.W., Zhang F.Y., Yan X.F., Zhang X.F., Dong Z.D., Cui D.Q., Chen F. (2017). Genome-wide association study for 13 agronomic traits reveals distribution of superior alleles in bread wheat from the Yellow and Huai Valley of China. Plant Biotechnol. J..

[28] Ellis M.H., Rebetzke G.J., Azanza F., Richards R.A., Spielmeyer W. (2005). Molecular mapping of gibberellin-responsive dwarfing genes in bread wheat. Theor. Appl. Genet..

[29] Piñera-Chavez F.J., Berry P.M., Foulkes M.J., Sukumaran S., Reynolds M.P. (2021). Identifying quantitative trait loci for lodging-associated traits in the wheat doubled-haploid population Avalon × Cadenza. Crop Sci..

[30] Cui F., Zhao C., Ding A., Li J., Wang L., Li X., Wang H. (2014). Construction of an integrative linkage map and QTL mapping of grain yield-related traits using three related wheat RIL populations. Theor. Appl. Genet..

[31] Dreccer M.F., Macdonald B., Farnsworth C.A., Paccapelo M.V., Awasi M.A., Condon A.G., Forrest K., Lee Long I., McIntyre C.L. (2022). Multi-donor× elite-based populations reveal QTL for low-lodging wheat. Theor. Appl. Genet..

[32] Song P., Wang X., Wang X., Zhou F., Xu X., Wu B., Yao J., Lv D., Yang M., Song X. (2021). Application of 50K chip-based genetic map to QTL mapping of stem-related traits in wheat. Crop Pasture Sci..

[33] Blackburn A., Sidhu G., Schillinger W.F., Skinner D., Gill K. (2021). QTL mapping using GBS and SSR genotyping reveals genomic regions controlling wheat coleoptile length and seedling emergence. Euphytica.

[34] Zhang D., Zhang X., Xu W., Hu T., Ma J., Zhang Y., Hou J., Hao C., Zhang X., Li T. (2022). TaGW2L, a GW2-like RING finger E3 ligase, positively regulates heading date in common wheat (*Triticum aestivum* L.). Crop J..

[35] Sánchez-Martín J., Widrig V., Herren G., Wicker T., Zbinden H., Gronnier J., Spörri L., Praz C.R., Heuberger M., Kolodziej M.C. (2021). Wheat Pm4 resistance to powdery mildew is controlled by alternative splice variants encoding chimeric proteins. Nat. Plants.

[36] Singh K., Saripalli G., Gautam T., Prasad P., Jain N., Balyan H.S., Gupta P.K. (2022). BS-Seq reveals major role of differential CHH methylation during leaf rust resistance in wheat (*Triticum aestivum* L.). Mol. Genet. Genomics..

[37] Agarwal P., Kapoor S., Tyagi A.K. (2011). Transcription factors regulating the progression of monocot and dicotseed development. Bioessays.

