# Supplementary material for: Genetic Analysis of Adaptive Traits in Spring Wheat in Northeast China
Source: Life (Basel). 2024 Jan 24;14(2):168. doi: 10.3390/life14020168 (PMC10890535; doi:10.3390/life14020168)
Supplement: Supplementary file 1 [file life-14-00168-s001.zip › Table S3.pdf]

Table S3 The primers of the *Kasp_2D_PH* and *Kasp_6D_HD*

SNP marker	KASP marker	Physical position (Mb) [a]	Primer name	Sequence (5'to 3') [b]
AX-111096297	*Kasp_2D_PH*	33.0	FAM	**GAAGGTGACCAAGTTCATGCT** ATAGCTCTGGCACTGCACCG
			HEX	**GAAGGTCGGAGTCAACGGATT** ATAGCTCTGGCACTGCACCC
			Common	TCAGGACAGACCAAGGAGATT
AX-110918412	*Kasp_6D_HD*	464.9	FAM	**GAAGGTGACCAAGTTCATGCT**ACGAGCCGTTGTACTTATAATGGA
			HEX	**GAAGGTCGGAGTCAACGGATT**ACGAGCCGTTGTACTTATAATGGC
			Common	TGGTATTACGAGGGCCGAAC

[a] Physical position (Mb) were obtained by blasting SNP flanking sequences against the Chinese Spring RefSeq v1.1 (https://urgi.versailles.inra.fr/blast_iwgsc/).

[b] FAM and HEX tails used for KASP marker assays are bolded.
