# Supplementary figures and images for: Genetic Analysis of Adaptive Traits in Spring Wheat in Northeast China
Source: Life (Basel). 2024 Jan 24;14(2):168. doi: 10.3390/life14020168 (PMC10890535; doi:10.3390/life14020168)

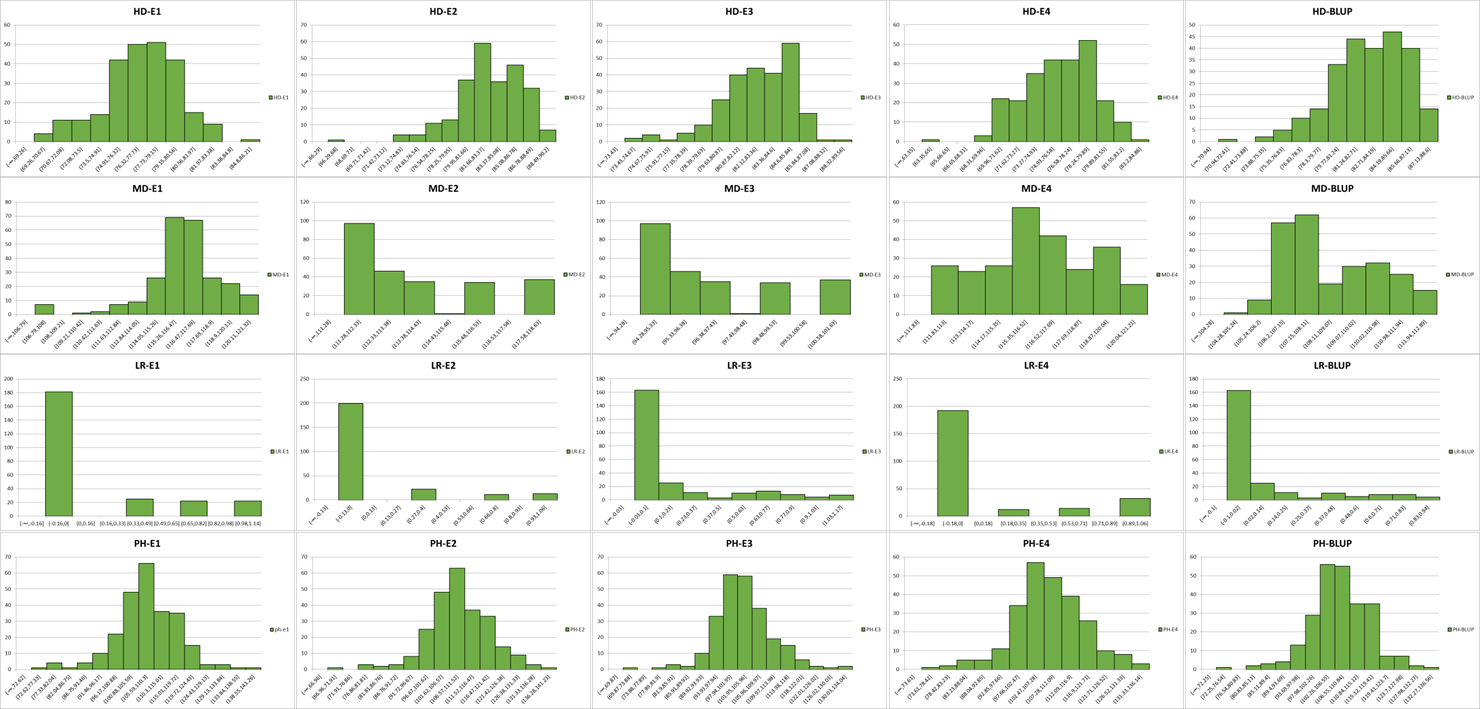

Supplement: Supplementary file 1 [file life-14-00168-s001.zip › Fig. S1.tif]

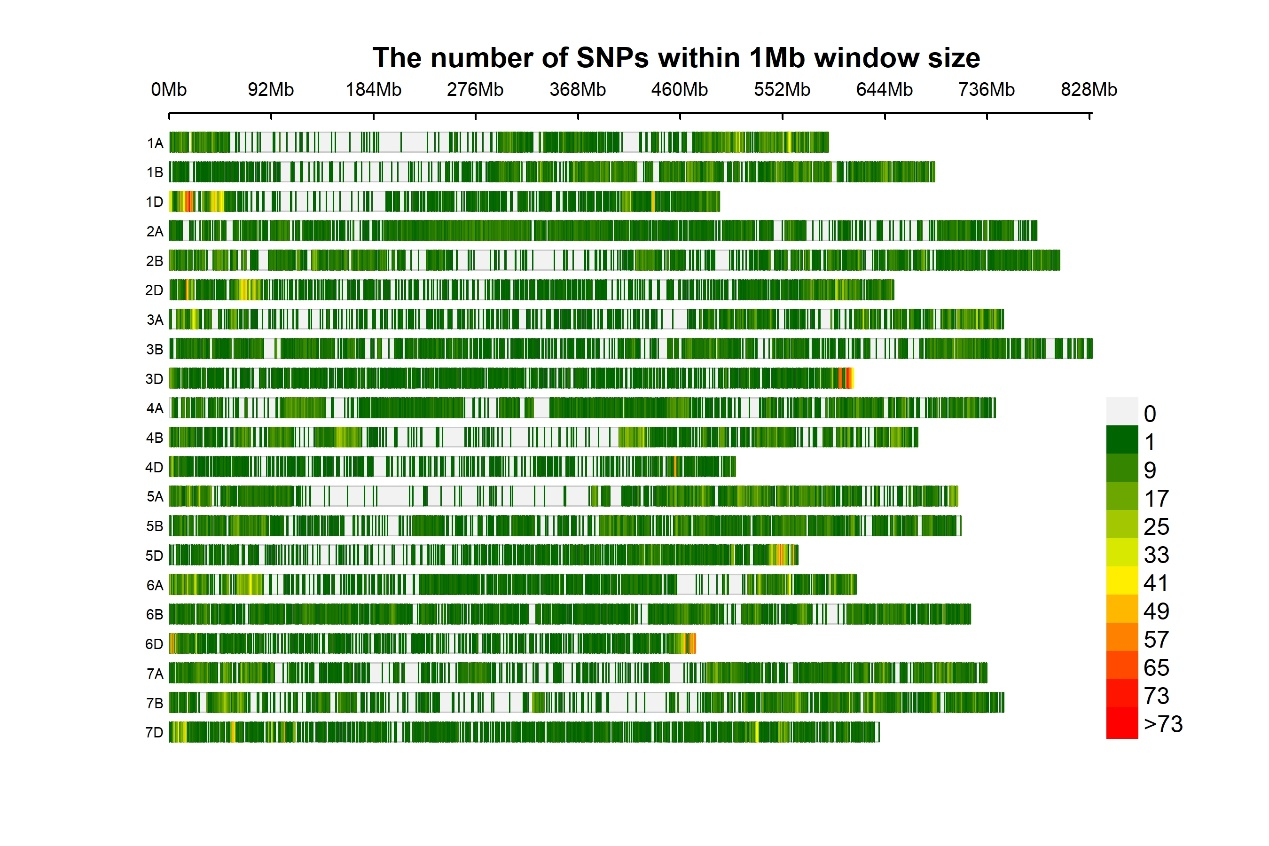

Supplement: Supplementary file 1 [file life-14-00168-s001.zip › Fig. S2.tif]

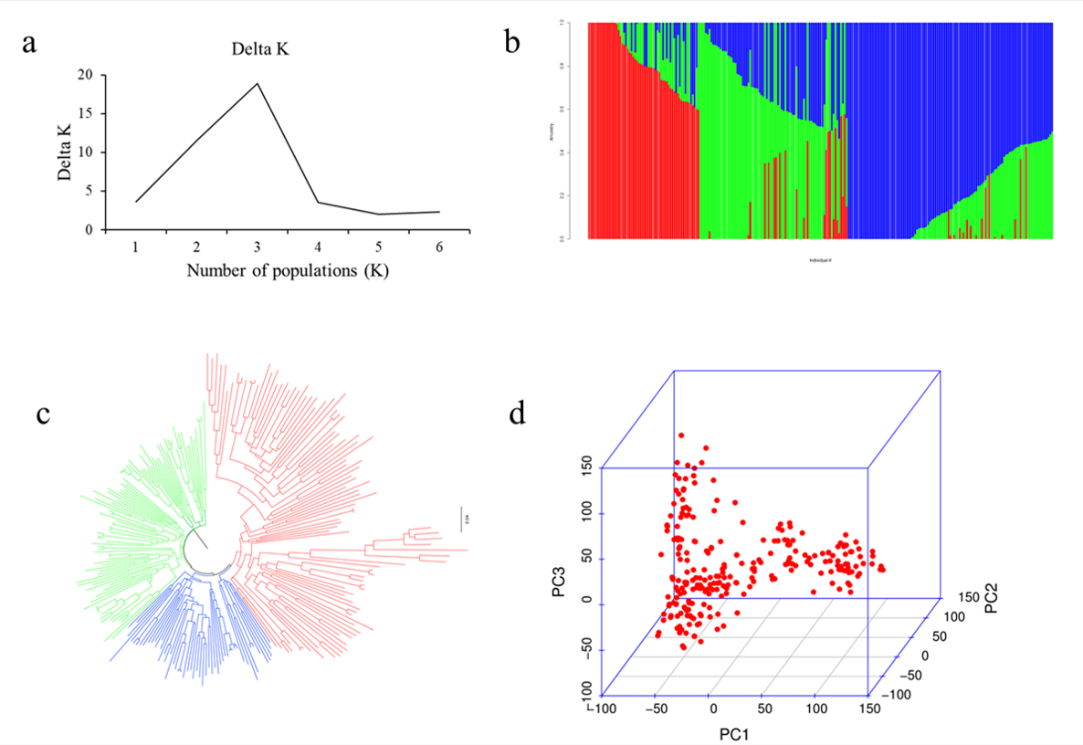

Supplement: Supplementary file 1 [file life-14-00168-s001.zip › Fig. S3.tif]

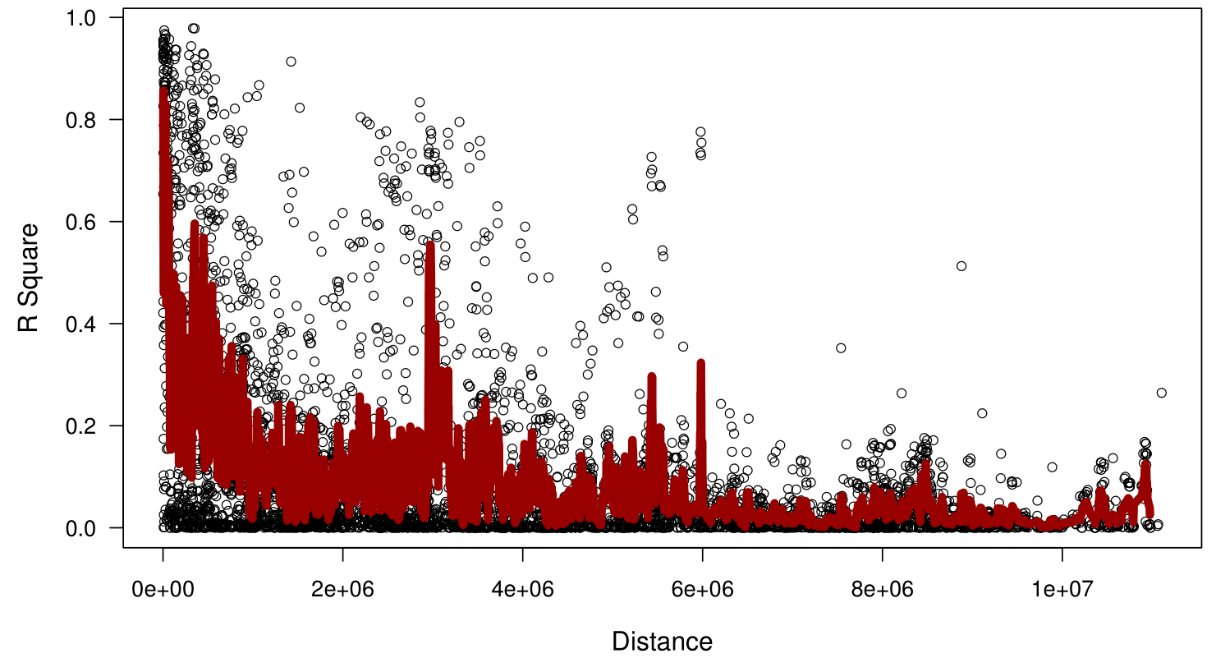

Supplement: Supplementary file 1 [file life-14-00168-s001.zip › Fig. S4.tif]

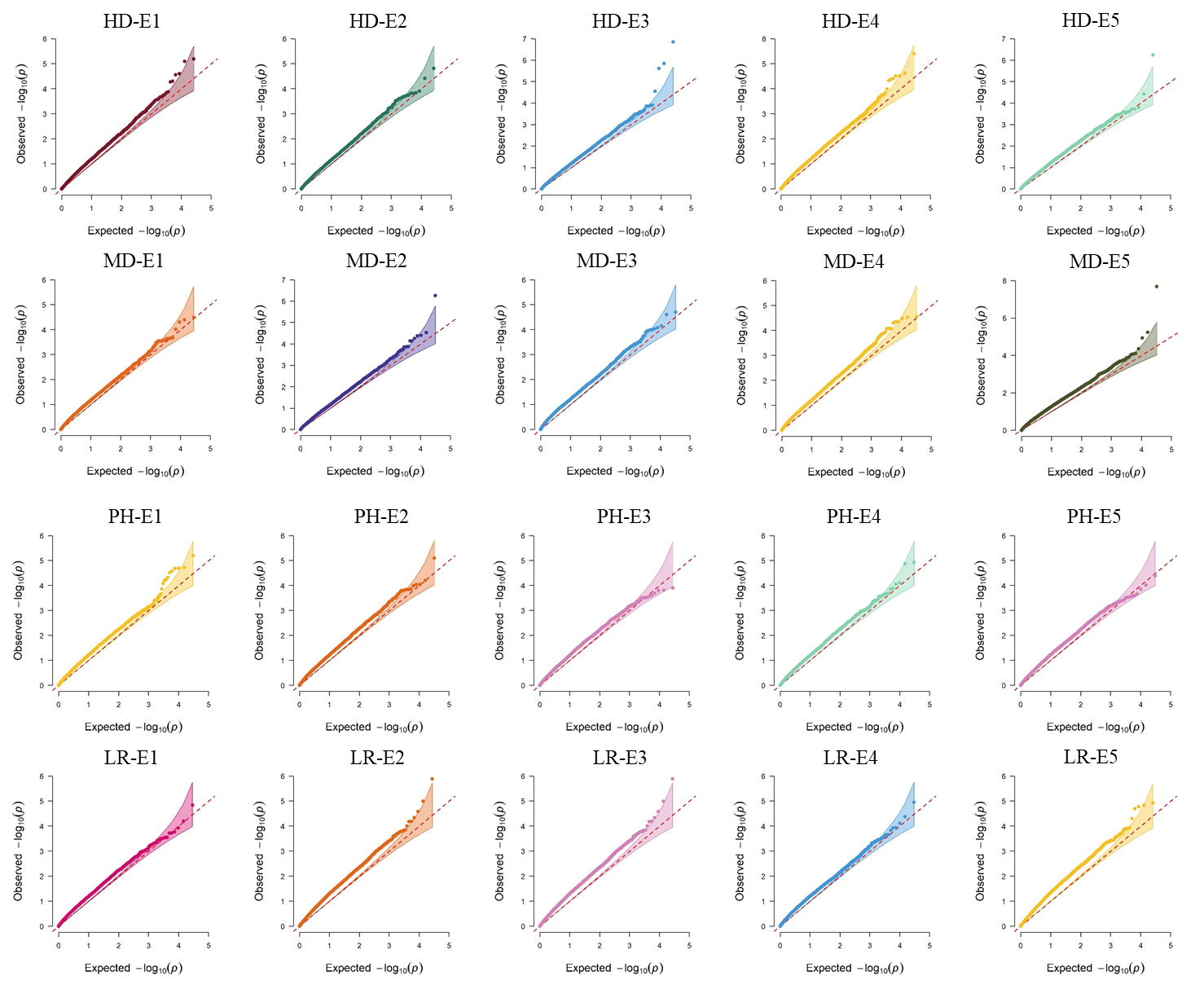

Supplement: Supplementary file 1 [file life-14-00168-s001.zip › Fig. S5.tif]
